# Cytochalasin D restores nuclear size acting on F-actin and IZUMO1 localization in low-quality spermatozoa

**DOI:** 10.7150/ijbs.77166

**Published:** 2023-04-17

**Authors:** Guillaume Martinez, Donato Cappetta, Marialucia Telesca, Konrad Urbanek, Giuseppe Castaldo, Magali Dhellemmes, Vincenza Grazia Mele, Teresa Chioccarelli, Veronica Porreca, Anne-Laure Barbotin, Angèle Boursier, Florian Guillou, Charles Coutton, Sophie Brouillet, Antonella De Angelis, Liberato Berrino, Riccardo Pierantoni, Gilda Cobellis, Rosanna Chianese, Francesco Manfrevola

**Affiliations:** 1 Hôpital Couple-Enfant, Centre Hospitalier Universitaire de Grenoble, UM de Génétique Chromosomique, 38000 Grenoble, France.; 2 Genetic Epigenetic and Therapies of Infertility, Institute for Advanced Biosciences INSERM U1209, CNRS UMR5309, 38000 Grenoble, France.; 3 Department of Experimental Medicine, University of Campania L. Vanvitelli, via Costantinopoli 16, 80138, Naples, Italy.; 4 Department of Biological and Environmental Sciences and Technologies, University of Salento, Lecce.; 5 Department of Molecular Medicine and Medical Biotechnologies, University of Naples “Federico II”, Via A. Pansini 5, 80131 Naples, Italy.; 6 CEINGE-Advanced Biotechnologies, Via G. Salvatore 486, 80131 Naples, Italy.; 7 CHU Lille, Institute de Biologie de la Reproduction-Spermiologie-CECOS, F-59000, Lille, France.; 8 CNRS, IFCE, INRAE, Université de Tours, PRC, Nouzilly, France.; 9 Université de Montpellier, EmbryoPluripotency, DEFE, INSERM 1203, Hôpital Arnaud de Villeneuve, CHRU Saint-Eloi, 80 Avenue Augustin Fliche, CEDEX 05, 34295 Montpellier, France.

**Keywords:** F-actin, IZUMO1, sperm quality, chromosomes territories, histone acetylation

## Abstract

In spermatozoa, the nuclear F-actin supports the acroplaxome, a subacrosomal structure involved in the correct exposure of several acrosomal membrane proteins; among them, the glycoprotein IZUMO1 is the major protein involved in sperm-oocyte fusion. Nuclear F-actin is also involved in sperm head shaping and chromosome compartmentalization.

To date, few notions regarding the bivalent role of F-actin on sperm chromatin organization and IZUMO1 positioning have been reported. In our work, we characterized subcellular organization of F-actin in human high- and low-quality spermatozoa (A- and B-SPZ), respectively, showing that F-actin over-expression in sperm head of B-SPZ affected IZUMO1 localization. A correct IZUMO1 repositioning following in *vitro* induction of F-actin depolymerization, by cytochalasin D treatment, occurred. Interestingly, F-actin depolymerization was also associated with a correct acrosome repositioning, thus to favor a proper acrosome reaction onset, with changes in sperm nuclear size parameters and histone acetylation rate reaching high-quality conditions.

In conclusion, the current work shows a key role of F-actin in the control of IZUMO1 localization as well as chromatin remodeling and acetylation events.

## Introduction

In mammalian spermatozoa, actin represents the most abundant cytoskeletal protein localized in sperm head which actively participates in several structural and biochemical functions such as: i) head shaping occurring during spermiogenesis, ii) sperm capacitation and iii) acrosome reaction (AR) 1-4. During spermiogenesis, sperm cell undergoes a massive morphological modification needful for fertility ability acquisition. This critical process includes a drastic nuclear condensation leading to the typical elongated head shape of spermatozoa 5-7. In particular, sperm head shaping, that coincides with acrosome biogenesis process, is supported by the acroplaxome, a nucleo-skeletal structure which not only anchors the acrosome to the nuclear envelope, ensuring its correct positioning, but also provides a mechanical scaffold plate during the nuclear shaping of the spermatid. Indeed, this scaffold plate modulates the exogenous forces that the F-actin-containing hoops of adjacent Sertoli cells apply to the elongating spermatid head in order to ensure a correct morphological nuclear maturation 8-9. Therefore, it is highly defined that acrosome formation assumes a central role in sperm head shaping, since its aberrations cause abnormal head morphology responsible for infertile spermatozoa production 8. Additional sperm functions are regulated by F-actin dynamics, as a fine temporal balance between polymerization and depolymerization of F-actin filaments occurs during capacitation and AR 1, 2. Indeed, during capacitation, a prominent polymerization of G-actin monomers to form F-actin filaments occurs in order to: i) prevent spontaneous AR in sperm head, ii) support spermatic hyperactivated motility (HAM) acquisition at flagellum level, whereas a rapid F-actin depolymerization occurs when AR starts 1.

The acrosome, that covers the anterior half part of sperm head as a cap structure, supports the sperm-oocyte interaction, through the AR, and exhibits a repertoire of proteins that mediate fertilization 8, 10. Among them, IZUMO1, a membrane glycoprotein localized in the acrosome, has attracted the attention 11. Indeed, *Izumo1^-/-^* male mice are infertile due to the failure of sperm-oocyte membrane interaction 12. However, in order to ensure sperm-oocyte fusion, IZUMO1 undergoes post-translational modifications, in particular phosphorylation, dependent on the activity of TSSK6, a testis-specific serine kinase, which regulates, *via* actin polymerization dynamics, the redistribution of IZUMO1 along the acrosome 13, 14. In this scenario, a contribution of F-actin is not to be excluded as in mice the inhibition of F-actin polymerization negatively affects IZUMO1 redistribution 13.

Additional roles of F-actin in several nuclear functions, such as chromatin organization and remodeling, are well reported 14. In the nucleus of eukaryotic cells, the chromatin is not randomly distributed since each individual chromosome occupies a specific position known as chromosome territory 15. This intricate chromosome compartmentalization ensures a proper regulation of gene replication, transcription as well as gene silencing 16 and, more interestingly, is driven by the nuclear actin organization. Indeed, chromosome movement and positioning are regulated by two different actin-dependent chromatin remodeling mechanisms: i) in the first, the direct interaction between monomeric actin and chromatin regulates chromatin remodeling, whereas ii) in the second, the chromatin-complexes move along actin filaments to explicate their regulatory action on chromatin 17-20. In this context, human spermatozoa show a well-defined nuclear organization in which chromosome positioning results meticulously organized in distinct intranuclear territories 21, 11. In this intricate sub-nuclear sperm chromosome organization, the centromeres organize themselves in a compact central nuclear volume named chromocenter, while the telomeres are projected at the nuclear periphery 22-24.

In the current work, we provide a molecular and morphological analysis of F-actin in two different human sperm populations: high-quality spermatozoa (arbitrarily called A-SPZ) and low-quality spermatozoa (arbitrarily called B-SPZ), collected from normozoospermic patients. If on one hand A-SPZ show normal morphology, good motility and the absence of damaged DNA, on the other hand B-SPZ display several anomalies such as low motility, morphological defects and DNA damage 25-27. Our investigations highlight an intriguing over-expression of F-actin in B-SPZ, preferentially at sperm head level. Based on the functional relationship between actin polymerization and IZUMO1 acrosomal distribution, we investigated the expression levels and localization of IZUMO1, as well as of TSSK6 kinase, in both A- and B-SPZ, showing a clear mis-localization of both in B-SPZ. IZUMO1 distribution anomalies were dependent on a strongly physical interaction with F-actin. Accordingly, depolymerization of F-actin, induced by *in vitro* Cytochalasin-D (CYTO-D) treatment, restored the normal IZUMO1 and TSSK6 localizations, as well as IZUMO1 phosphorylation rate, in B-SPZ. Interestingly, F-actin depolymerization in B-SPZ also restored the normal acrosome positioning to favor AR onset, and thus IZUMO1 physiological redistribution.

In addition, we demonstrated increased nuclear size in B- compared to A-SPZ that was surprisingly restored to physiological values by CYTO-D treatment. Based on these intriguing observations, we decided to investigate chromosomal territories in sperm populations pre- and post CYTO-D treatment, in order to evaluate a putative anomalous chromosome remodeling in low-quality sperm population dependent on F-actin overexpression. No differences were observed in chromosomal territories between A- and B-SPZ, but a strong histone acetylation rate in B-SPZ, restored to physiological state following CYTO-D treatment, was detected. All together our data show, for the first time, how the over-expression of F-actin in the head of B-SPZ negatively affects IZUMO1 localization. Interestingly, our experimental approach based on CYTO-D treatment was able to restore IZUMO1 localization as well as nuclear size and histone acetylation rate, leading us to assert that IZUMO1 represents a gold standard marker not only to select good spermatozoa useful for fertilization, but also to identify the spermatozoa with the optimal nuclear size as well as the correct chromatin folding dependent on histone acetylation state.

## Material and Methods

### Ethical Approval

In accordance with the Declaration of Helsinki, sperm samples were obtained from normozoospermic volunteers patients after their written informed consent. This study involving human participants was reviewed and approved by the ethics committee of Azienda Sanitaria Locale (ASL) Caserta, Regione Campania (n. 1353, 27 October 2017). All patients were interviewed in order to better understand their area of origin, their eating habits, as well as their lifestyles.

### Human Semen Collection and Spermatozoa Isolation by Density Gradient Centrifugation

Semen samples collected from normozoospermic (n=15) volunteers, were produced by masturbation after 5-7 days of sexual abstinence and collected in sterile sample containers. After liquefaction for 30 min at 37°C, semen parameters, such as concentration, total motility, progressive motility and morphology were evaluated by using computer-assisted sperm analysis (CASA) technology associated with the Sperm Class Analyzer (SCA) system (SCA version 6.1; Microptic, S.L. Viladomat, Barcelona, Spain), in accordance to the WHO reference criteria. Human A- and B- quality SPZ fractions were purified by using density gradient centrifugation approach, as previously reported 26. In brief, 1 mL of human semen sample was loaded at the top of 40/80% discontinuous PureCeption (Cooper Surgical, Trumbull, CT, United States) gradient and centrifuged at 300 × g for 20 min. Following centrifugation, A-SPZ fraction was purified from 80% PureCeption, placed at the base of conical plastic tube 30 mm in diameter, while B-SPZ fraction was purified from 40% PureCeption placed at the top of 80% PureCeption. Then, the two sperm fractions were washed once with 10 mL of sperm washing medium (HTF-IrvineScientific^®^) to remove the PureCeption and centrifuged at 500 × g for 15 min.

Following centrifugation, an aliquot of the sample was used to evaluate the number of live SPZ under a light microscope, by using the viable dye Trypan-blue, in order to exclude any died cell contaminations (data not shown). Then, sperm samples were treated in ice for 30 min with Somatic Cell Lysis Buffer (SCLB) (0.1% SDS, 0.5% Triton X-100 in DEPC-H_2_O) to eliminate any somatic cell contamination. Following the SCLB treatment, an aliquot of sample was used to verify the elimination of somatic cells, by using microscope examination, and to confirm that the density gradient procedure did not change motility and morphological sperm parameters, by repeating CASA technology. Then, A- and B-SPZ fractions were resuspended in sperm washing medium (HTF-IrvineScientific^®^) and counted under a light microscope, using a Burker Chamber, in order to pellet equal concentrations of both fractions (1 × 10^7^ cells) for molecular investigations. Sperm pellets were stored at -80°C. Aliquots of A- and B-SPZ were dried on slides and properly stored at -20°C for immunofluorescence analysis.

### Experimental animals

C57BL/6 male mice (Charles River Laboratory, Lecco, Italy) were used in this study. All animals were housed under controlled illumination (12 h light/dark cycle; light on 6:00 AM) and standard environmental conditions (ambient temperature 20-22°C, humidity 55-60%) and were maintained on a standard pellet diet with free access to water. The number of the enrolled animals was determined by the parameters adopted for the G*Power analysis, required to get the permission for *in vivo* experiments, which is suggested by the Legal Entity giving the permission. For experimental procedures, adult males (3-5 months) were sacrificed under anaesthesia, by cervical dislocation. Animals were placed in a plexiglass chamber with 4% isoflurane (Iso-Vet, Piramal Healthcare, UK Limited) for 5 min and were sacrificed when fully sedated, as measured by a lack of heartbeat and active paw reflex.

Experiments involving animals were approved by the Italian Ministry of Education and the Italian Ministry of Health, with authorization n°405/2021-PR. Procedure involving animal care were carried out in accordance with the National Research Council's publication Guide for Care and Use of Laboratory Animals (National of Institutes of Health Guide).

### Mouse sperm collection from *caput* and *cauda* epididymis

*Caput* and* cauda* epididymis (from n=6 mice) were separately immersed in PBS (pH 7.6) and cut to let SPZ flow out from the ducts. Samples were then filtered throughout cheesecloth to eliminate fragments of epididymal tissue and centrifuged at 1500 × g for 30 min at 4°C. After centrifugation, the SPZ pellets were incubated on ice for 30 min with Somatic Cell Lysis Buffer (SCLB; 0.1% SDS, 0.5% Triton X-100 in DEPC-H_2_O) to eliminate possible contamination by somatic cells. After lysis, SPZ were centrifuged at 800 × g for 15 min at 4°C and then washed twice with PBS. Aliquots of *caput* and *cauda* SPZ were both stored at -80°C for protein extraction and dried on slides to be finally stored at -20°C for immunofluorescence analysis.

### CYTO-D *in vitro* treatment of human SPZ

CYTO-D, a potent inhibitor of F-actin polymerization, was obtained from Sigma-Aldrich (C8273; Milan, Italy). The drug was dissolved in dimethylsulfoxide (DMSO) according to the manufacturer's instructions. Human A- and B-SPZ pellets (1 x 10^7^ cells) collected from normozoospermic volunteers were purified as described above and incubated in PBS (1 ml) for 30 min at 37°C with vehicle [0.005% DMSO; CTRL groups (pre-A; pre-B)] or with CYTO-D at 10 μM [experimental groups (post-A; post-B)]. CYTO-D dose and time of incubation were chosen in order not to affect cell viability on the base of previous data 27. Following treatment, an aliquot of the sample was used to evaluate the number of live SPZ under a light microscope, by using the viable dye Trypan-blue, thus to exclude any harmful effect on cell viability (data not shown). In addition, an aliquot of sample was used to assess changes in sperm motility parameters, by repeating CASA analysis. After that, sperm samples were centrifuged at 1500 × g for 20 min at 4°C and washed twice with PBS. Sperm pellet was properly stored at -80°C for molecular investigations or used to collect sperm head enriched-fractions as following reported. Aliquots of sperm samples were dried on slides and properly stored at -20°C for immunofluorescence analysis.

### Separation of Sperm into Head-Enriched Fraction

The sperm pellets (1 x 10^7^ cells) collected from CTRL (pre-A; pre-B) and SPZ *in vitro* treated with CYTO-D (10 μM) (post-A; post-B) were used to have sperm head enriched-fractions as reported by Kichine *et al* 28. In brief, sperm samples were resuspended in PBS pH 7.6 and sonicated at 4°C, 3 × 20 sec at 20 Hz, using Soniprep150 Ultrasonic Disintegrator (SANYO Electric Co. Ltd., Japan) to break off sperm tails into small pieces. Following, the samples were centrifuged at 9300 × g for 15 min. The tail pieces were recovered in the supernatant, while a pellet of sperm heads was obtained at the bottom of the tube. Head and tail purity was examined by microscopic analysis. Sperm head pellets were stored at -80°C for following molecular investigations. Aliquot of sperm head samples was dried on slides and properly stored at -20°C for immunofluorescence analysis. Supernatants with sperm tail were processed by using our previously protocol 29. In brief, sonicated supernatants were layered over a discontinuous sucrose gradient containing 10 ml 0.9 M sucrose and 15 ml 2.0 M sucrose, prepared in Buffer B (0.01 M Tris-HCl, 0.001 MEDTA, pH 7.6). The gradients were centrifuged at 10000 rpm in a SW28 rotor in Beckman Optima L-90K Ultracentrifuge (Beckman Coulter, USA) at 10°C for 30 min. Sperm tails were collected from the interface of 0.9 M sucrose and 2.0 M sucrose and stored at -80°C for following molecular investigations.

### Protein extraction and western blot analysis

All the analyzed experimental groups [human normozoospermic A- and B-SPZ fractions; total SPZ or sperm heads and tails collected from CTRL (pre-A; pre-B) and SPZ *in vitro* treated with CYTO-D (10 μM) (post-A; post-B)] and murine SPZ collected from *caput* and *cauda* epididymis were separately homogenized in RIPA buffer [PBS, pH 7.4, 10 mM dithiothreitol, 0.02% sodium azide, 0.1% SDS, 1% NP-40, 0.5% sodium deoxycholate, in the presence of protease inhibitors (10 μg/ml of leupeptin, aprotinin, pepstatin A, chymostatin, and 5 μg/ml of TPCK)] and sonicated three times for 30 sec bursts, each at 60 mW. Proteins were separated by SDS-PAGE (4-20% Mini-PROTEAN® TGX™ Precast Protein Gels; 4561094, Bio-Rad Laboratories, Italy) and transferred to polyvinylidene difluoride membrane (GE Healthcare, Milano, Italy) at 280 mA for 2.5 h at 4°C. The filters were treated for 3 h with blocking solution [5% non-fat milk, 0.25% Tween-20 in Tris-buffered saline (TBS, pH 7.6)] and then separately incubated overnight, at 4°C in TBS-milk buffer (TBS pH 7.6, 3% non-fat milk) with different primary antibodies [IZUMO1 (ab211623) from Abcam, Cambridge, UK; beta-actin (PA1-16889) and F-actin (BS-1571R) from Invitrogen, Milano, Italy; p-Ser/Phosphoserine (sc-81514) Santa Cruz Biotechnology, Heidelberg, Germany; H4K5Ac (E-AB-66468), H4K8Ac (E-AB-67844) and H4K12Ac (E-AB-66253) from Elabscience, Texas, USA; histone H3 (ab1791) from Abcam, Cambridge, UK]. After washing in 0.25% Tween20-TBS, filters were incubated with 1:1000 horseradish peroxidase-conjugated rabbit IgG (Dako Corp., Milano, Italy) in TBS-milk buffer and then washed again. The immune complexes were detected using the enhanced chemiluminescence-western blotting detection system [Amersham ECL western Blotting Detection Reagent (RPN2106) GE Healthcare, Milano, Italy]. Signals were quantified by densitometry analysis, adjusted relatively to Pon.S and graphed as OD values or fold change (mean ± SEM).

### Protein immunoprecipitation (IP)

For IP, 1 x 10^7^ heads and/or total human normozoospermic A- and B-SPZ fractions, collected from CTRL (pre-A; pre-B), human SPZ *in vitro* treated with CYTO-D (10 μM) (post-A; post-B) and murine SPZ collected from *caput* and *cauda* epididymis, were lyzed with RIPA buffer, in the presence of protease inhibitors (10 μg/ml of leupeptin, aprotinin, pepstatin A, chymostatin, and 5 μg/ml of TPCK) and sonicated three times for 30 sec bursts, each at 60 mW. After sonication, the samples were incubated on ice for 30 min and centrifuged at maximum speed for 30 min 4°C. Then, a concentration of 500 mg of supernatant proteins from each sample was incubated with 2 μg of relative antibody [IZUMO1 (ab211623) from Abcam, or IgG (12370; Sigma-Aldrich, Milano, Italy)] under rotary agitation at 4°C overnight. Following, Protein A/G PLUS Agarose Beads (sc-2003; Santa Cruz Biotechnology, Heidelberg, Germany) were added to each sample for 4 h, at 4°C under rotary agitation and then washed 3 times (3000 × g for 3 min a 4°C) in 500 µl of cold TBS pH 7.6. The samples were boiled in Laemmli sample buffer for 10 min to be later analyzed by SDS-PAGE in comparison to related Input controls.

### Human Sperm Immunofluorescence and 3-D imaging

Human normozoospermic A- and B-SPZ fractions, SPZ and/or heads collected from CTRL (pre-A; pre-B) and SPZ *in vitro* treated with CYTO-D (10 μM) (post-A; post-B) were dried on slides as above reported and fixed in 4% paraformaldehyde (sc-281692; Santa Cruz Biotechnology, Heidelberg, Germany) for 20 min at RT and then permeabilized with 0.1% Triton X-100 (X100; Sigma-Aldrich, Milano, Italy). Blocking was carried out with 10% of donkey serum (ab7475; Abcam, Cambridge, UK) for 30 min at RT and then cells were separately incubated with different primary antibodies [IZUMO1 (ab211623) from Abcam; PNA (L21409) from Invitrogen, Milano, Italy; TSSK6 (sc-514076) from Santa Cruz Biotechnology, Heidelberg, Germany] overnight at 4°C. Following three washes in DPBS (1X), a fluorescein isothiocyanate (FITC) conjugated was used as secondary antibody (711-095-152; Jackson ImmunoResearch, Cambridge, UK) for 1 h at 37°C. Nuclei were labeled with DAPI (D9542; Sigma-Aldrich, Milano, Italy) while F-actin was labeled with phalloidin (21834; Thermo Fisher Scientific, USA). All samples were analyzed with a Zeiss LSM700 confocal microscope. Immunofluorescence experiments were performed in triplicate. For the fluorescent signal analysis, 20 fields/sample were analyzed. Densitometric analysis of immunofluorescence was performed with ImageJ Software (version 1.53 g) and adjusted relatively to DAPI fluorescence intensity.

For IZUMO1, an isotype control by using the same isotype (IgG) at the same concentration of IZUMO1 primary antibody [Rabbit IgG, polyclonal - Isotype Control (ab37415) from Abcam, Cambridge, UK] and an isoclonic control by using IZUMO1 peptide [Recombinant Human IZUMO1 protein (ab127522) from Abcam, Cambridge, UK] were carried out.

For optical sectioning and 3-D imaging, a confocal laser scanning microscope Leica TCS-SP8 STED with a HC PL APO CS2 63x/1.4 oil immersion objective was used. The confocal pinhole was set to 1 Airy to optimize z-sectioning. Images and z-stacks were recorded sequentially with a step size of 300 nm. For histone acetylation, SPZ from A- and B-SPZ fractions, CTRL (pre-A; pre-B) and SPZ *in vitro* treated with CYTO-D (10 μM) (post-A; post-B) were incubated with different primary H4Ac antibodies separately [H4K5Ac (E-AB-66468), H4K8Ac (E-AB-67844) and H4K12Ac (E-AB-66253) from Elabscience, Texas, USA] and processed as above reported. A Rhodamine Red™-X (RRX) conjugated was used as secondary antibody (711-295-152; Jackson ImmunoResearch, Cambridge, UK) for 1 h at 37°C. Nuclei were labeled with DAPI (D9542; Sigma-Aldrich, Milano, Italy) and the analysis was carried out under an optical microscope (Leica DM 5000 B + CTR 5000) with a UV lamp. Densitometric analysis of immunofluorescence was performed with ImageJ Software (version 1.53 g) and adjusted relatively to DAPI fluorescence intensity.

### Murine Sperm Immunofluorescence

Murine SPZ collected from *caput* and *cauda* epididymis were dried on slides as above reported and fixed in 4% paraformaldehyde (sc-281692; Santa Cruz Biotechnology, Heidelberg, Germany) for 20 min at RT and then permeabilized with 0.2% Triton X-100 (X100; Sigma-Aldrich, Milano, Italy). Blocking was carried out with 10% of donkey serum (ab7475; Abcam, Cambridge, UK) for 30 min at RT and then cells were separately incubated with different primary antibodies [IZUMO1 (ab211623) from Abcam Cambridge, UK; PNA (L21409) from Invitrogen, Milano, Italy; TSSK6 (sc-514076) from Santa Cruz Biotechnology, Heidelberg, Germany] overnight at 4°C. Following three washes in DPBS (1X), a fluorescein isothiocyanate (FITC) conjugated was used as secondary antibody (711-095-152; Jackson ImmunoResearch, Cambridge, UK) for 1 h at 37°C. Nuclei were labeled with DAPI (D9542; Sigma-Aldrich, Milano, Italy), while F-actin was labeled with phalloidin (21834; Thermo Fisher Scientific, USA). All samples were analyzed under an optical microscope (Leica DM 5000 B + CTR 5000) with a UV lamp. Densitometric analysis of immunofluorescence was performed with ImageJ Software (version 1.53 g) and adjusted relatively to DAPI fluorescence intensity.

### Transmission electron microscopy (TEM)

TEM experiments were performed using human A- and B-SPZ ± CYTO-D (pre-A, pre-B, post-A and post-B). Pellets were first fixed in 2.0% v/v glutaraldehyde in phosphate buffer (pH 7.4), then washed in fresh buffer with 4% w/v sucrose for 15 min, and finally embedded in 2% agar. Post-fixation was performed using 1% osmic acid in phosphate buffer. Agar-embedded samples were then dehydrated in successive graded ethanol baths. After dehydration, small pieces of agar containing SPZ were further embedded in Epon resin (Polysciences). Sections were cut on a Reichert OmU2 ultramicrotome (Reichert-Jung AG) with a diamond knife. Ultrathin sections (70 nm) were collected on Parlodion 0.8%/isoamyl acetate-coated 100 mesh Nickel grids (EMS, Fort Washington, PA) and counterstained with 2% uranyl acetate and lead citrate before observation. Sections were examined with a Zeiss transmission electron microscope 902 (Leo, Rueil-Malmaison, France). Images were acquired using a Gatan Orius SC1000 CCD camera (Gatan France, Grandchamp, France).

### Sperm nuclear area analysis

PFA-fixed SPZ were spread on slides and nuclei were stained with DAPI II solution (Abbott Laboratories, Chicago, USA). Nuclear area was precisely evaluated by NMAS according to the analysis method previously described 30 using images of the DAPI-stained nuclei. Nucleus detection settings were: Kuwahara kernel: 3, and flattening threshold: 100, for preprocessing; canny low threshold: 0.5, canny high threshold: 1.5, canny kernel radius: 3, canny kernel width: 16, gap closing radius: 5, for objects finding; min area: 1 000, max area: 10 000, min circ: 0.1, max circ: 0.9, for filtering. Sperm nuclear analysis was performed using NMAS software (version 1.19.2, https://bitbucket.org/bmskinner/nuclear_morphology/wiki/Home) and images were obtained with a Zeiss Imager Z2 microscope, using a CoolCube 1 CCD camera, with a 100x/1.4 Zeiss objective and Neon software (MetaSystems, Altlussheim, Germany).

### Sperm hybridization *In-Situ* Fluorescence analysis (FISH)

Carnoy solution-fixed sperm was spread on slides and processed according to standard FISH procedures as previously described 31 with a modified decondensation step. Briefly, samples were washed in 2× of SSC (saline-sodium citrate) solution and decondensed with a 30 min exposition to a Dithiothreitol solution (10mM DTT in 0.1M Tris-HCl pH8 with 0.1% Triton X-100). Samples were then washed again in 2× of SSC, dehydrated and hybridized with specific house-made or commercial probes according to manufacturer's protocol in a HYBrite system (Abbott Laboratories). Sperm nuclei were counterstained with a DAPI II solution (Abbott Laboratories, Chicago, USA).

For chromosome territory size and position analysis, samples were hybridized with MetaSystems whole-chromosome painting probes XCP 9 (D-0309), XCP 11 (D-0311), XCP 17 (D-0317), XCP 21 (D-0321), XCP 22 (D-0322), XCP X (D-0323). For intertelomeric analysis, samples were hybridized with homemade probes built as described previously 32 with 9p probe consisting of 5 bacterial artificial chromosome clones (RP11-996P21, RP11-675G5, RP11-696A8, RP11-960F12 and CTD-2537D15) covering a 1 Mb distance, and a 9q probe consisting of 8 bacterial artificial chromosome clones (RP11-937L7, RP11-417A4, RP11-48C7, CTD-2377P2, RP11-350O14, RP11-673E5, RP11-769N4 and CTD-2551F21) covering a 1.2 Mb distance. For chromocenter analysis, samples were hybridized with Pan-centromeric probe (1695-F-01 from Cambio, Cambridge, England).

### Sperm chromatin parameters interpretation analysis

Image analysis was performed according to the method described in Skinner et al. 2019 33. Briefly, detection settings used for nuclear signals acquisition were: Threshold: 70, Min size: 5, Max fraction: 1, Min circ: 0, Max circ: 1. For intertelomeric distance analysis, distance between signal pairs (both telomere signals) was evaluated in microns by the colocalization function of the software. For chromosome territories, area was provided in microns post nuclear signals detection, radial position was evaluated using a 3-layer shell from the shells function, and linear position was evaluated using a simple linear filter. For chromocenter analysis, we used a multi-scale structural similarity index measure (MS-SSIM) to compare signal distributions and quantifies visual similarities between the populations 34 after warping of the different sperm populations signals on a sperm consensus of all sperm populations.

### Sperm chromatin integrity assays

Human normozoospermic A- and B-SPZ fractions were used for Chromomycin A3 (CMA3) and Aniline blue staining, to evaluate protamination and histone rates, respectively. In brief, for CMA3 staining sperm cells were fixed in methanol/acetic solution (n=4 different samples for each experimental group) and spread on Superfrost slides air-dried at RT overnight. Cells were subsequently incubated in 0.25 mg/ml of CMA3 solution in McIlvaine buffer (pH 7) for 20 min and washed twice for 2 min in McIlvaine buffer. Slides were then mounted with DAPI II (Abbott Laboratories) and a minimum of 200 cells were evaluated for each sample. For Aniline blue staining, sperm cells were fixed in 3% glutaraldehyde solution and dried on slides. Slides were hydrated 5 min in water, stained in 5% aniline blue solution diluted in 4% acetic acid solution, rinsed twice for 2 min in water, dehydrated through ethanol bath series (70, 90, and 100% ethanol solutions), and finally fixed in toluene for 2 min. Then, slides were mounted with Eukitt^®^. At least 200 cells were evaluated with a Nikon Eclipse 80i epifluorescence microscope with a 100X oil objective. All assays were performed on four different replicates (n=4) for both A- and B-SPZ.

### Sperm functional parameters assays

Human normozoospermic A- and B-SPZ collected from CTRL (pre-A; pre-B) and following *in vitro* CYTO-D treatment (post-A; post-B) were used for *in vitro* AR induction and capacitation state assessment as previously reported 35. In brief, for AR induction SPZ treated with CYTO-D were incubated with 10 μM of Ca^2+^ ionophore A23187 (C7522; Sigma-Aldrich, Milano, Italy) for 1 h at RT. After incubation, SPZ were dried on slides and stored for following PNA and IZUMO1 immunofluorescence analyses.

For capacitation assessment, human normozoospermic A- and B-SPZ fractions from CTRL (pre-A; pre-B) and following *in vitro* CYTO-D treatment (post-A; post-B) were stained with a chlortetracycline (CTC) solution contained 750 μmol of CTC I-1 (26430; Sigma-Aldrich, Milano, Italy) in a buffer containing 130 mmol NaCl, 5 mmol cysteine and 20 mmol Tris-HCl. After incubation, sperm cells were fixed in PFA 4%, dried on slides and analyzed under an optical microscope (Leica DM 5000 B + CTR 5000).

### Statistical Analysis

ANOVA followed by Student's t-test (for two independent group comparisons) and Tukey test (for multi group comparison) were used to identify groups having different mean. Differences with p<0.05 were considered statistically significant. Data were expressed as the mean ± SEM from at least five independent samples for each experimental group. For Western blot analysis, triplicates from five experimental groups were considered.

For sperm nuclear area and chromosome territory size and position analyses, Mann-Whitney U tests with Bonferroni multiple testing correction were automatically calculated by the software. P-values were considered significant when equal or inferior to 0.05. If not stated otherwise, all other data were treated with R software (version 3.5.2). Histograms display mean ± standard deviation (SD) and statistical significance of differences was assessed by applying an unpaired Student t-test. P-values were considered significant when equal or inferior to 0.05.

## Results

### F-actin overexpression associates with IZUMO1 mis-localization in human low-quality SPZ

**Figure 1:**
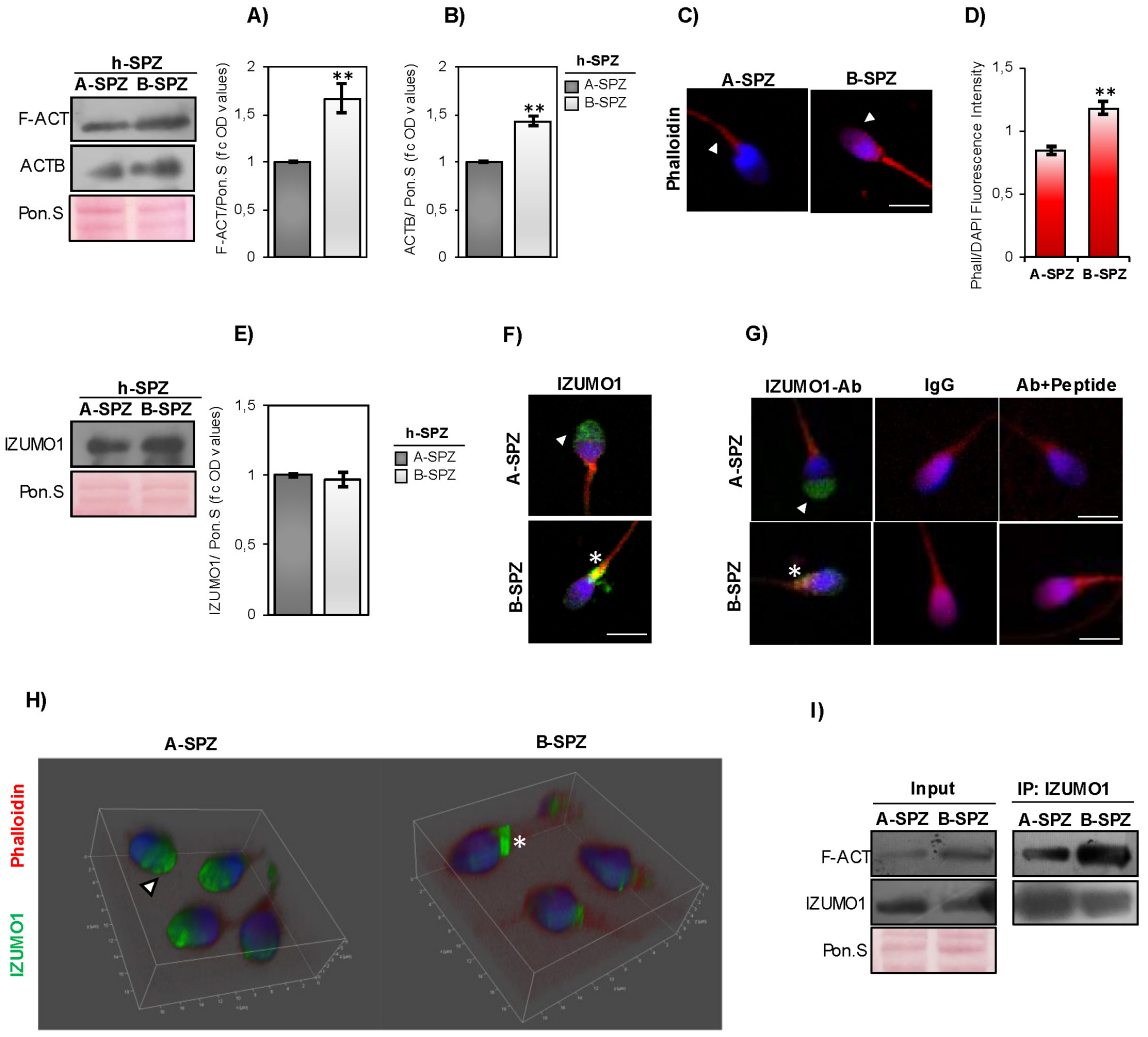
** F-actin and IZUMO1 characterization in human high- and low-quality spermatozoa. (A-B)** Western blot analysis of (**A**) F-actin (F-ACT) and (**B**) beta-actin (ACTB) proteins in A- and B-SPZ fractions collected from normozoospermic volunteers (n=6 different samples in triplicate for each sperm fraction). Signals were quantified by densitometry analysis and normalized to Ponceau Red (Pon.S). Data were expressed in OD values as fold change and reported as mean ± SEM; **p < 0.01. (**C**) F-actin immunofluorescence analysis by phalloidin staining (red) in A- and B-SPZ fractions collected from normozoospermic volunteers (n=6 different samples for each sperm fraction). White arrowheads represent F-actin localization in sperm head. Nuclei were labeled with DAPI (blue). Scale bar corresponds to 4 μm. (**D**) Histogram showing the quantification of phalloidin fluorescence signal intensity using ImageJ software. Values were expressed as mean ± SEM, **: p<0.01 (n=6 different samples in triplicate for each sperm fraction). (**E**) Western blot analysis of IZUMO1 protein in A- and B-SPZ fractions collected from normozoospermic volunteers (n=6 different samples in triplicate for each sperm fraction). Signals were quantified by densitometry analysis and normalized to Ponceau Red (Pon.S). Data were expressed in OD values as fold change and reported as mean ± SEM. (**F**) Immunofluorescence analysis of IZUMO1 (FITC-green) in A- and B-SPZ fractions collected from normozoospermic volunteers (n=6 different samples for each sperm fraction). White arrowheads represent acrosomal IZUMO1 localization in sperm head of A-SPZ; white asterisk represents IZUMO1 localization in the neck-midpiece of B-SPZ. Nuclei were labeled with DAPI (blue), while F-actin was labeled with phalloidin (red). Scale bar corresponds to 4 μm. (**G**) Immunofluorescence analyses of Ab-IZUMO1 (FITC-green), rabbit IgG (FITC-green) and Ab-IZUMO1+IZUMO1-peptide (FITC-green) in A- and B-SPZ. White arrowheads represent sperm head acrosomal localizations; white asterisks represent localizations in sperm head posterior part and the apical part of midpiece. Nuclei were labeled with DAPI (blue), while F-actin was labeled with phalloidin (red). Scale bar corresponds to 4 μm. (**H**) Confocal z-stack 3-D reconstruction strategy showing the head (white arrowheads) *vs* neck-midpiece (white asterisks) localization of IZUMO-1 (green). (**I**) Western blot analysis of protein immunoprecipitation assay (IP) in A- and B-SPZ using IZUMO1 antibody. IZUMO1-IP was analyzed in comparison with Input protein extracts.

In order to investigate a differential F-actin content in human high- and low-quality SPZ (arbitrarily called A- and B-SPZ, respectively), western blot analysis was carried out between the two different sperm populations collected by density gradient procedure, as previously reported 26. As showed, the F-actin content was significantly higher in B- than A-SPZ (p<0.01) (Fig. 1A). A significant increase of beta-actin was found in the same sperm population (p<0.01) (Fig. 1B), suggesting that the enhancing of actin filament formation in B-SPZ was probably due to the increase of actin monomer. With the aim to clarify putative differences in the subcellular localization of F-actin, phalloidin staining was carried out in A- and B-SPZ, respectively. Data showed, in both sperm populations, a bivalent F-actin localization in sperm head and tail (Fig. 1C). In addition, F-actin signal was more intense in B-SPZ sperm head when compared to A-SPZ population (Fig. 1C), whereas fluorescence intensity analysis confirmed F-actin overexpression in B-SPZ as observed by western blot analysis (Fig. 1D).

IZUMO1 protein content was investigated in A- and B-SPZ by western blot analysis. No quantitative difference was observed between the two sperm populations (Fig. 1E). Based on this, immunofluorescence analysis was carried out in A- and B-SPZ with the aim to investigate possible anomalies of IZUMO1 localization in human low-quality SPZ. As showed, in A-SPZ the IZUMO1 localization was closely related to the anterior half part of sperm head (Fig. 1F), at acrosome level, whereas in B-SPZ the localization was completely reversed as IZUMO1 signal appeared in the posterior half part of sperm head, related to the implantation fossa and the apical part of midpiece, likely related to sperm neck-midpiece (Fig. 1F).

To confirm that the peculiar IZUMO1 signal observed in B-SPZ was not dependent on non-specific interactions of IZUMO1 primary antibody, we carried out two selective immunofluorescence controls: firstly, we set an isotype control incubating the samples with a non-immune antibody of the same isotype (IgG) and at the same concentration of IZUMO1 primary antibody. As second control, we mixed IZUMO1 primary antibody with an excess amount of IZUMO1 peptide against which the primary antibody is directed, in order to block primary antibody binding-sites and perform an isoclonic control. As showed, all experimental groups lost IZUMO1 signal (Fig. 1G), confirming the specificity of IZUMO1 observed in A- and B-SPZ. In agreement, confocal X-Z optical sectioning following 3-D reconstruction granted the head-neck distinction further supporting the qualitative difference and altered subcellular localization of IZUMO1 and thus confirming the signal in the posterior half part of sperm head of B-SPZ (Fig. 1H).

In order to shed light on a possible protein interaction between F-actin and IZUMO1, immunoprecipitation experiments (IP) were carried out in A- and B-SPZ by using IZUMO1 antibody. As reported, IZUMO1-IP showed a protein signal for F-actin in both sperm populations, stronger in B- than in A-SPZ (Fig. 1I), suggesting a tighter interaction between the two proteins in human low-quality sperm population.

### F-actin depolymerization drives IZUMO1-TSSK6 sperm head distribution

**Figure 2:**
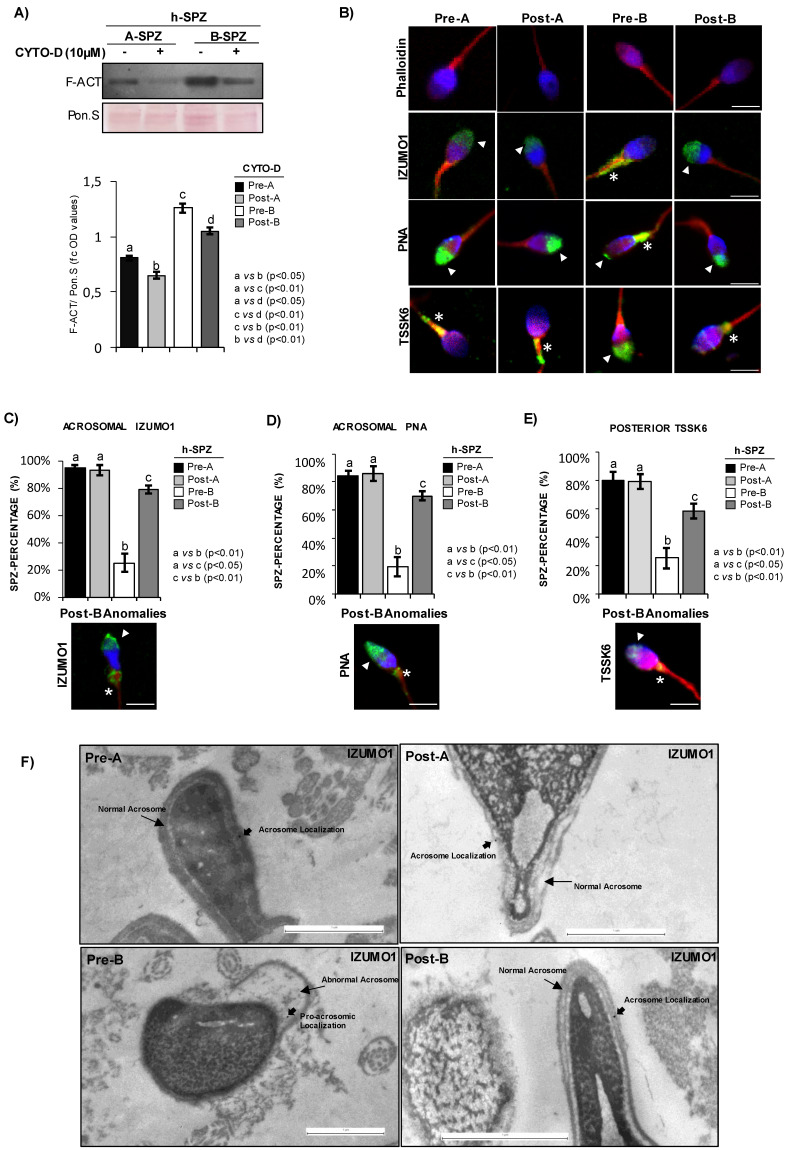
** F-actin depolymerization restores acrosomal IZUMO1 localization.** (**A**) Western blot analysis of F-actin (F-ACT) in A- and B-SPZ fractions before (pre-A; pre-B) and following *in vitro* CYTO-D (post-A; post-B) (n=6 different samples for each experimental group in triplicate). Signals were quantified by densitometry analysis and normalized to Ponceau Red (Pon.S). Data were expressed in OD values as fold change and reported as mean ± SEM. Experimental groups with statistically significant differences (p<0.05; p<0.01) were indicated with different letters. (**B**) Phalloidin staining (red) and immunofluorescence analyses of IZUMO1 (FITC-green), PNA (FITC-green) and TSSK6 (FITC-green) in A- and B-SPZ fractions before (pre-A; pre-B) and following *in vitro* CYTO-D treatment (post-A; post-B) (n=6 different samples for each experimental group). White arrowheads represent sperm head acrosomal localizations; white asterisks represent localizations in sperm head posterior part. Nuclei were labeled with DAPI (blue), while F-actin was labeled with phalloidin (red). Scale bar corresponds to 4 μm.** (C-E)** Cellular counting of sperm cells having (**C**) typical IZUMO1 acrosomal localization, (**D**) typical PNA acrosomal localization and (**E**) typical TSSK6 posterior localization in A- and B-SPZ fractions before (pre-A; pre-B) and following *in vitro* CYTO-D treatment (post-A; post-B) (n=6 different samples in triplicate for each experimental group; more than 100 cells were analyzed for each sample). Data were expressed as percentage of positive cells on total and reported as mean ± SEM. Experimental groups with statistically significant differences (p<0.05; p<0.01) were indicated with different letters; the experimental groups without statistically significant differences were indicated with the same letter. Images of IZUMO1, PNA and TSSK6 anomalies in post-B were showed. (**F**) Transmission Electron Microscopy (TEM) experiments in A- and B-SPZ treated with CYTO-D (pre-A; pre-B; post-A; post-B). Short black arrows represent IZUMO1 localization, while long black arrows indicate acrosome position. Scale bar corresponds to 1 μm.

In order to investigate if F-actin directly affects IZUMO1 localization, *in vitro* CYTO-D experiments were carried out in both A- and B-SPZ. Western blot analysis confirmed a significant reduction of F-actin content, following CYTO-D treatment, in both sperm populations (post-A; post-B) in comparison to the respective control ones (pre-A; pre-B) (Fig. 2A). Interestingly, CYTO-D treatment did not restore F-actin levels of post-B experimental group to pre-A physiological values (Fig. 2A). Phalloidin staining showed a reduction of F-actin signal, following CYTO-D treatment, closely related to sperm tail and head, in post-A and post-B SPZ, respectively (Fig. 2B).

Accordingly, IZUMO1 localization in A-SPZ (post-A) was not affected by CYTO-D treatment, whereas a redistribution of IZUMO1 from the sperm neck-midpiece at the base of the head to the anterior half part of sperm head, at acrosomal level, was observed in post-B SPZ (Fig. 2B), reinforcing the hypothesis of a putative nuclear F-actin primary role on IZUMO1 positioning. In order to show that IZUMO1 redistribution observed in post-B SPZ was not dependent on acrosomal alteration induced by CYTO-D treatment, acrosomal status was evaluated by immunofluorescence analysis using peanut lectin PNA antibody. As showed, PNA signal was well confined only to acrosomal region in pre-A and post-A experimental groups, confirming that CYTO-D treatment did not affect the acrosomal area. Surprisingly, a redistribution of PNA signal from the posterior to the anterior half part of sperm head, similar to IZUMO1 one, was observed in post-B SPZ (Fig. 2B), suggesting that abnormal IZUMO1 localization observed in B-SPZ could be dependent on aberrant F-actin content in sperm head and, in turn, on an anomalous acrosome positioning. Finally, immunofluorescence analysis of TSSK6 kinase, a member of TSSK kinase family essential for the proper IZUMO1 localization 12, 13, showed a clear TSSK6 localization in the posterior part of sperm head, related to the apical part of midpiece, in pre-A and post-A SPZ (Fig. 2B), whereas an atypical acrosomal localization was observed in pre-B SPZ (Fig. 2B). Accordingly, CYTO-D pharmacological treatment in B-SPZ (post-B) reverted TSSK6 localization at neck-midpiece level (Fig. 2B), suggesting that physiologically TSSK6 and IZUMO1 have opposite specular localization.

With the aim to quantify the effects of CYTO-D treatment on IZUMO1 redistribution observed in post-B sperm population, a sperm count was carried out in all experimental groups using the acrosomal IZUMO1 localization as morphological parameter of good sperm quality. Accordingly to the previous data, the percentage of sperm cells having an acrosomal IZUMO1 localization did not change between pre- and post-A experimental groups, whereas a significant increase was observed in post-B compared to pre-B experimental group (Fig. 2C). Interestingly, CYTO-D treatment was not able to restore the percentage of sperm cells having acrosomal IZUMO1 localization to physiological values as a small percentage of post-B SPZ showed an incomplete apical IZUMO1 relocation (Fig. 2C). Accordingly, sperm count using the acrosomal PNA localization as inclusive analysis parameter showed the same trend (Fig. 2D). Interestingly, the sperm count using the posterior TSSK6 localization as morphological parameter of good sperm quality showed an incomplete TSSK6 redistribution in post-B SPZ following CYTO-D treatment, similarly to IZUMO1 and PNA observed trends (Fig. 2E).

In order to thoroughly investigate the anomalous position of IZUMO1 highlighted in pre-B SPZ by immunofluorescence and 3-D analyses, we performed TEM experiments in all experimental groups by using IZUMO1 antibody (Fig. 2F). As reported, in pre-A, post-A and post-B SPZ a clear intact acrosome, composing of continue outer and inner acrosomal membrane, was observed. As expected, in these experimental groups, IZUMO1 showed a typical anterior acrosomal localization at membrane level (Fig. 2F). Interestingly, most sperm of pre-B experimental group displayed an atypical acrosome with abnormal structure and positioning, characterized by an abnormal shape likely due to an incorrect movement of pro-acrosomic vesicles to the anterior part of the head. Consistently IZUMO1 lost its localization at acrosomal membrane level in pre-B SPZ (Fig. 2F), confirming immunofluorescence data and the anomalous acrosome maturation, as well as positioning, highlighted by PNA staining in the same sperm population.

### IZUMO1 maturation during murine epididymal transit

**Figure 3:**
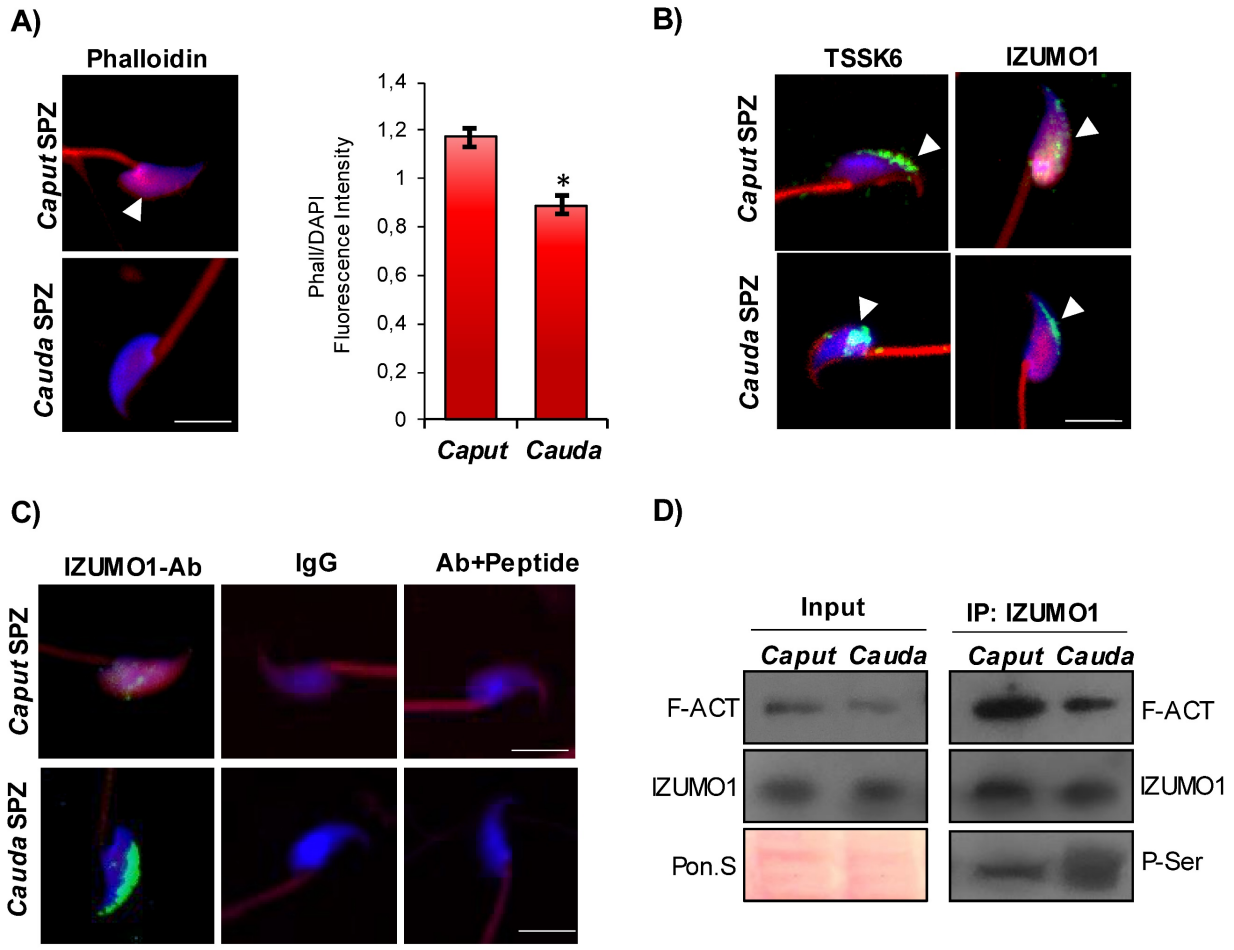
** Characterization of IZUMO1 maturation during epididymal transit.** (**A**) F-actin immunofluorescence analysis by phalloidin staining (red) in murine SPZ collected from *caput* and *cauda* epididymis (n=6 different samples for each experimental group). White arrowheads represent F-actin localization in sperm head. Nuclei were labeled with DAPI (blue). Scale bar corresponds to 4 μm. (**B**) Immunofluorescence analysis of IZUMO1 (FITC-green) and TSSK6 (FITC-green) in murine SPZ collected from *caput* and *cauda* epididymis (n=6 different samples for each experimental group). White arrowheads represent IZUMO1 and TSSK6 localizations in sperm head. Nuclei were labeled with DAPI (blue), while F-actin was labeled with phalloidin (red). Scale bar corresponds to 4 μm. (**C**) Immunofluorescence analyses of Ab-IZUMO1 (FITC-green), rabbit IgG (FITC-green) and Ab-IZUMO1+IZUMO1-peptide (FITC-green) in murine SPZ collected from *caput* and *cauda* epididymis. Nuclei were labeled with DAPI (blue) while F-Actin was labeled with phalloidin (red). Scale bar corresponds to 4 μm. (**D**) Western blot analysis of protein immunoprecipitation assay (IP) in *caput* and *cauda* SPZ using IZUMO1 antibody. IZUMO1-IP was analyzed in comparison with Input protein extracts.

In order to shed light on the intriguing interaction among F-actin, IZUMO1 and TSSK6 kinase, we decided to analyze these actors in murine SPZ during their physiological epididymal maturation, with the idea that immature murine SPZ collected from *caput* epididymis could mimic human low-quality SPZ. Phalloidin staining carried out on the epididymal SPZ showed a significant reduction of F-actin signal, related to sperm head, from *caput* to *cauda* as confirmed by fluorescence intensity analysis (Fig. 3A). Immunofluorescence analysis of TSSK6 kinase showed in *caput* sperm head a clear localization in the anterior half part of sperm head, at acrosomal level, more similar to the localization observed in human B-SPZ, whereas a translocation to the posterior part of sperm head was detected in *cauda* SPZ (Fig. 3B). Interestingly, IZUMO1 showed a diffuse localization in *caput* sperm head that was progressively well confined to the acrosomal region in *cauda* SPZ (Fig. 3B). As previously reported, an immunofluorescence isotype and isoclonic control for IZUMO1 was carried out, and the specify of signal was confirmed (Fig. 3C). Since IZUMO1 maturation during epididymal transit consists in a strong phosphorylation wave 12, 13, we decided to carry out IP using IZUMO1 antibody in *caput* and *cauda* SPZ, separately, in order to investigate a possible differential interaction rate among F-actin and IZUMO1 during epididymal transit, able to regulate IZUMO1 phosphorylation state. Western blot analysis showed a decrease of F-actin-IZUMO1 interaction from *caput* to *cauda* dependent on variations of F-actin content during epididymal transit, as confirmed by the analysis on input samples (total lysates isolated before the IP) (Fig. 3D). IZUMO1 levels were constant from *caput* to *cauda,* as confirmed by western blot analysis of input samples, whereas a significant increase of IZUMO1 phosphorylation was observed in *cauda* SPZ as suggested by the increase of phospho-serine signal (P-Ser) in IP (Fig. 3D), suggesting that physiologically the loss of F-actin-IZUMO1 interaction enhanced TSSK6 nuclear redistribution and, in turn, IZUMO1 phosphorylation.

### F-actin depolymerization drives AR in B-SPZ

**Figure 4:**
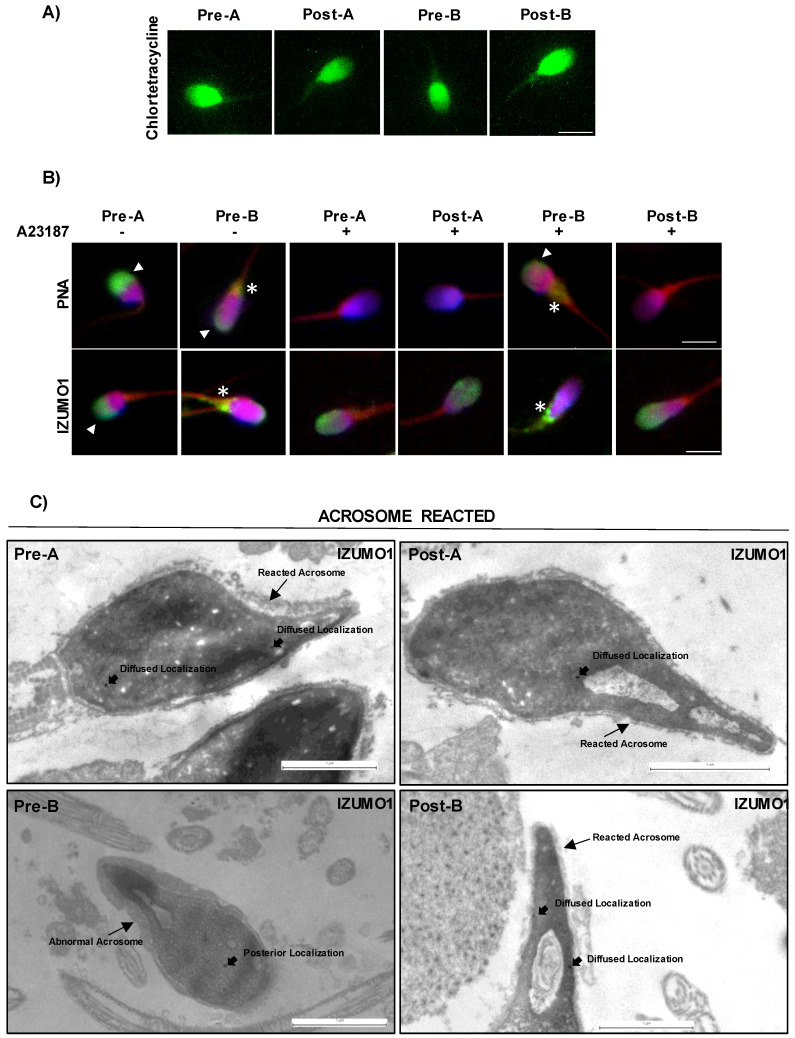
** Characterization of CYTO-D effects on sperm functional parameters.** (**A**) Sperm capacitation analysis by chlortetracycline, CTC (green) staining in A- and B-SPZ before (pre-A; pre-B) and following *in vitro* CYTO-D treatment (post-A; post-B) (n=4 different samples for each experimental group). Scale bar corresponds to 4 μm. (**B**) Immunofluorescence analyses of PNA (FITC-green) and Ab-IZUMO1 (FITC-green) in A- and B-SPZ treated with CYTO-D (pre-A; pre-B; post-A; post-B) following AR induced by A23187. White arrowheads represent sperm head acrosomal localization of PNA and IZUMO1; white asterisks represent localizations in sperm neck-midpiece. Nuclei were labeled with DAPI (blue), while F-actin was labeled with phalloidin (red). Scale bar corresponds to 4 μm. (**C**) TEM experiments in A- and B-SPZ treated with CYTO-D (pre-A; pre-B; post-A; post-B) following AR induction. Short black arrows represent IZUMO1 localization, while long black arrows indicate acrosome position. Scale bar corresponds to 1 μm.

Considering that F-actin dynamics underlie several sperm functional parameters, we investigated the effects of CYTO-D treatment on sperm motility, capacitation and AR, in order to shed a light on possible functional restoring effects on B-SPZ. Firstly, we carried out motility analysis on A- and B-SPZ in both physiological conditions and following CYTO-D treatment by using CASA system, in order to assess if F-actin dynamic could be able to modulate sperm motility parameters. As reported in Table 1, the progressive motility and the main motility parameters (VCL; VSL; VAP; LIN; STRA) were significantly reduced (p<0.01) in B- when compared to A-SPZ. Then, we investigated spermatic motility following CYTO-D treatment and a significant reduction of all parameters investigated was observed in post-A SPZ relatively to pre-A control group (Table 2). Conversely, motility parameters of post-B were slightly affected by CYTO-D treatment, relatively to pre-B control ones, confirming the selective F-actin modulation tightly related to sperm head instead of tail, as previously suggested by phalloidin staining. Considering that HAM acquisition during sperm capacitation is dependent on flagellar F-actin polymerization onset, we assessed sperm capacitation following CYTO-D treatment by CTC immunofluorescence staining. As expected on motility data above reported, CTC staining showed the typical pattern of non-capacitated sperm, consisting of full head signal, in all experimental groups (pre-A; pre-B; post-A; post-B), confirming that F-actin depolymerization contrasted *in vitro* sperm capacitation (Fig. 4A).

In order to investigate a putative effect on AR, exerted by CYTO-D treatment *via* F-actin modulation, we set a key experiment. Firstly, we carried out CYTO-D treatment on A- and B-SPZ, as previously reported, and then we induced *in vitro* AR, by using Ca^2+^ ionophore, in all experimental groups (pre-A; pre-B; post-A; post-B). Following treatments, PNA and IZUMO1 were investigated by immunofluorescence analysis. As showed, following AR the PNA signal disappeared in both pre- and post-A SPZ while IZUMO1 showed a more diffused head localization as compared to untreated A-SPZ, suggesting that CYTO-D treatment did not affect AR and the related IZUMO1 redistribution in A-SPZ (Fig. 4B). Interestingly, the same PNA null signal and IZUMO1 diffused head localization was observed in post-B SPZ but not in pre-B control group, that appeared similar to untreated B-SPZ. These data strongly suggested that CYTO-D treatment in B-SPZ, by modulating abnormal nuclear F-actin, induced a correct acrosome repositioning, as previously showed, in order to favor a correct AR onset and in turn IZUMO1 redistribution. To confirm this hypothesis, we performed TEM experiments on all experimental groups by using IZUMO1 antibody (Fig. 4C). Reacted acrosome following CYTO-D treatment of pre-A, post-A and post-B SPZ experimental groups, showed the typical disassembly of outer acrosome membrane occurring following AR, with small remaining parts observed. Interestingly, a similar pattern was not observed in pre-B SPZ, as acrosome structure and positioning appeared abnormal and not correctly shaped, strongly suggesting that AR onset was hampered. Regarding IZUMO1 distribution in pre- and post-A SPZ following AR, a clear diffused head localization occurred, confirming IZUMO1 immunofluorescence analysis above reported. Surprisingly, in reacted pre-B SPZ IZUMO1 localization was only confined to head posterior segment, whereas a diffused redistribution, similarly to pre- and post-A SPZ, occurred in reacted post-B SPZ (Fig. 4C), demonstrating that CYTO-D treatment in B-SPZ, *via* nuclear F-actin dynamics, modulated the proper pro-acrosomic vesicle anterior movement, and thus the acrosome positioning, to favor AR onset and consecutive IZUMO1 redistribution in all sperm head.

### CYTO-D treatment affects IZUMO1/F-actin interaction in sperm head of human B-SPZ enhancing IZUMO1 phosphorylation

**Figure 5:**
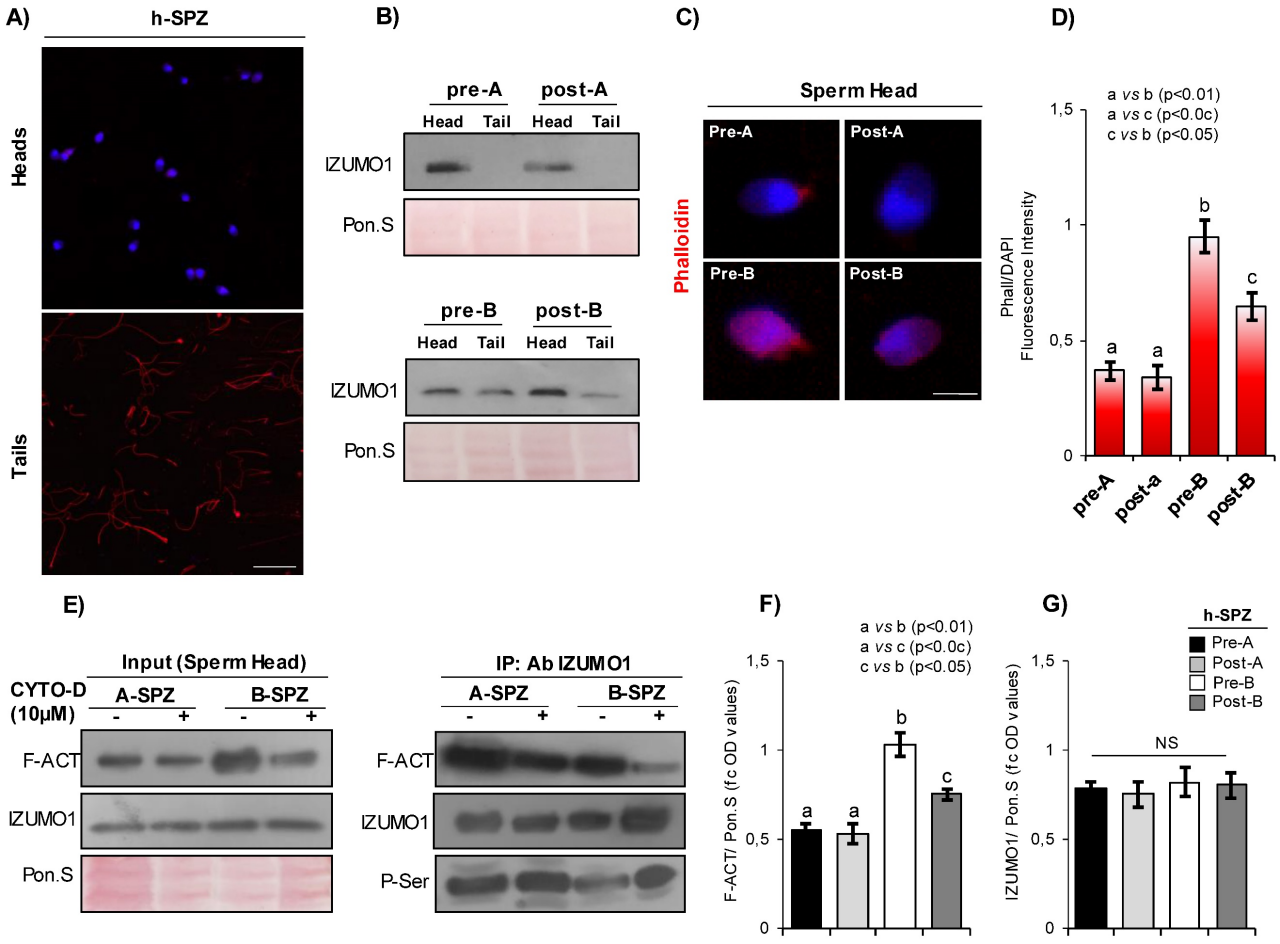
** Characterization of IZUMO1/F-actin interaction in human sperm head.** (**A**) Separation of A- and B-SPZ before (pre-A; pre-B) and following *in vitro* CYTO-D treatment (post-A; post-B) into head- and tail-enriched fractions. Representative images of sperm showing head and tail-enriched fractions, as indicated. Scale bar corresponds to 50 μm. (**B**) Western blot analysis of IZUMO1 protein in head- and tail-enriched protein fractions of A- and B-SPZ before (pre-A; pre-B) and following *in vitro* CYTO-D treatment (post-A; post-B) (n=3 different samples for each experimental group in triplicate) using IZUMO1 antibody. Signals were normalized to Ponceau Red (Pon.S). **(C)** F-actin immunofluorescence analysis by phalloidin staining (red) in head-enriched fractions of A- and B-SPZ before (pre-A; pre-B) and following *in vitro* CYTO-D treatment (post-A; post-B) (n=6 different samples for each experimental group). Nuclei were labeled with DAPI (blue). Scale bar corresponds to 4 μm.** (D)** Histogram showing the quantification of phalloidin fluorescence signal intensity using ImageJ software. Values were expressed as mean ± SEM, **: p<0.01 (n=6 different samples in triplicate for each sperm fraction). (**E**) Western blot analysis of protein immunoprecipitation assay (IP) in head-enriched fractions of A- and B-SPZ before (pre-A; pre-B) and following *in vitro* CYTO-D treatment (post-A; post-B) using IZUMO1 antibody. IZUMO1-IP was analyzed in comparison to Input protein extracts. (**F-G**) Signal quantification of (**F**) F-actin (F-ACT) and (**G**) IZUMO1 of Input protein extracts western blot analyses in head-enriched fractions of A- and B-SPZ before (pre-A; pre-B) and following *in vitro* CYTO-D treatment (post-A; post-B). Signals were quantified by densitometry analysis and normalized to Ponceau Red (Pon.S). Data were expressed in OD values as fold change and reported as mean ± SEM; experimental groups with statistically significant differences (p<0.05; p<0.01) were indicated with different letters; the experimental groups without statistically significant differences were indicated with the same letter.

In order to demonstrate that CYTO-D treatment preferentially induced F-actin depolymerization in the head of B-SPZ, enhancing in turn IZUMO1 phosphorylation, the heads of treated sperm cells (post-A; post-B) and of related control ones (pre-A; pre-B) were separated from tails and collected for immunofluorescence and molecular analyses (Fig. 5A).

Firstly, the heads and the tails of treated sperm cells (post-A; post-B) and related control ones (pre-A; pre-B) were processed for IZUMO1 western blot analysis. Results showed a clear absence of IZUMO1 in tails of pre- and post-A SPZ, while a strong content was observed in pre-B SPZ, which appeared significantly reduced in favor of respective head content following CYTO-D treatment, definitively confirming the anomalous localization of IZUMO1 in B-SPZ and the specificity of apical part of midpiece signal previously discovered (Fig. 5B). Phalloidin staining carried out on A- and B-SPZ, before (pre-A; pre-B) and following (post-A; post-B) CYTO-D treatment, respectively, showed a F-actin signal stronger in the head of B- than A-SPZ, whereas a significant F-actin depolymerization following CYTO-D treatment was observed only in the heads of post-B SPZ (Fig. 5C), as confirmed by fluorescence intensity analysis (Fig. 5D).

IP carried out using IZUMO1 antibody showed a clear decrease of F-actin signal in post-B experimental group, suggesting that F-actin depolymerization induced by CYTO-D treatment negatively affected the physical interaction between F-actin and IZUMO1 proteins in the sperm heads of B-SPZ (Fig. 5E). In addition, the loss of F-actin-IZUMO1 interaction enhanced IZUMO1 phosphorylation (Fig. 5E), as suggested by the increase of phospho-serine signal (P-Ser) in IP, reinforcing the idea that F-actin interaction negatively affected IZUMO1 functional maturation as well as its acrosomal positioning. As confirmed by the analysis on input samples (total lysates isolated before the immunoprecipitations), the reduction of F-actin-IZUMO1 interaction in post-B SPZ was dependent on variations of F-actin content, as a significant F-actin reduction (p<0.05), not completely restored to physiological pre-A values, was observed in heads derived from post-B SPZ (Fig. 5F). Accordingly, IZUMO1 levels were constant in all experimental groups*,* as confirmed by western blot analysis of input samples (Fig. 5G).

### CYTO-D treatment restored low-quality spermatozoa nuclear size to physiological rate

**Figure 6:**
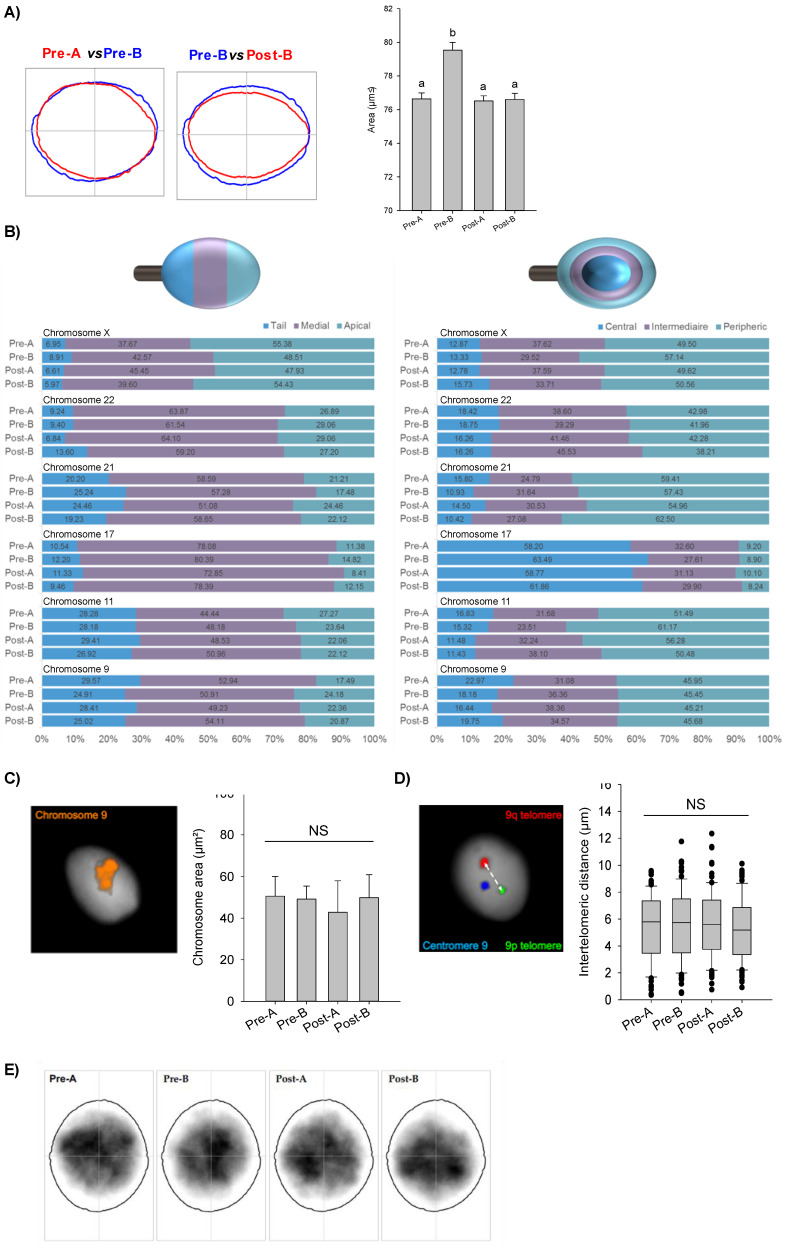
** F-actin depolymerization restores nuclear size of low-quality spermatozoa.** (**A**) Nuclear area analysis in A- and B-SPZ fractions before (pre-A; pre-B) and following *in vitro* CYTO-D treatment (post-A; post-B) (n=3 individuals for each experimental group; with more than 500 cells were analyzed per individual); graphical representation of nuclear consensus was obtained with NMAS software. (**B**) Frequency of linear and radial positioning within the sperm nucleus of territory of six different chromosomes territories in A- and B-SPZ fractions before (pre-A; pre-B) and following *in vitro* CYTO-D treatment (post-A; post-B) (n=4 individuals for each experimental group; with more than 400 nuclei were analyzed per individual);** (C-D)** Representative size of the territory and intertelomeric distance of chromosome 9 in sperm nuclei from A- and B-SPZ fractions before (pre-A; pre-B) and following *in vitro* CYTO-D treatment (post-A; post-B); (**E**) Heatmaps of chromocenter position in sperm nuclei from A- and B-SPZ fractions before (pre-A; pre-B) and following *in vitro* CYTO-D treatment (post-A; post-B) by NMAS.

In order to investigate a possible involvement of nuclear F-actin in chromosome movement and positioning, we performed a set of nuclear analyses looking at variations among CYTO-D treated sperm cells (post-A; post-B) and related control ones (pre-A; pre-B). We firstly used Nuclear Morphology Analysis Software (NMAS, with method described in detail in the relative section) to assess sperm nuclei size (Fig. 6A). Pre-A SPZ had an average area of 76.54 ± 1.05 µm², while pre-B SPZ had a statistically higher area of 81.38 ± 1.13 µm² (pre-A *vs* pre-B, n=5, Mann-Whitney U-test, U=93027, p<0.0001) (Table 3), suggesting an anomalous nuclear size in B-SPZ. Interestingly, CYTO-D treatment reduced the nuclear area of pre-B SPZ to 77,91 ± 0,98 µm², not statistically different from pre-A SPZ (post-B *vs* pre-A, n=5, Mann-Whitney U-test, U=110368, p>0.05), while it had no effect on the latter with a mean area of 77.70 ± 0.80 µm² for post-A SPZ (pre-A *vs* post-A, n=5, Mann-Whitney U-test, U= 145181, p>0.05) (Table 4).

Then, we hypothesized that the increase in nuclear volume of pre-B SPZ could be associated with an abnormal chromosomal distribution within nuclei. In sperm, chromosomes are non-randomly positioned within the nuclei and occupy domains with preferential radial and linear positioning called chromosomes territories (CT) 36, 37. We randomly chose and investigated six chromosomes in sperm nucleus: chromosomes 9, 11, 17, 21, 22 and X. All studied chromosomes displayed specific and non-random positioning (Pearson's Chi-squared test, p<0.01), but no differences (Pearson's Chi-squared test, p>0.05) were uncovered among the four sperm populations (Fig. 6B). Because chromosomal territories could be altered without their positions being impacted, we further investigated one of them. We randomly selected chromosome 9 and looked for possible variations in its territory size among the sperm populations (Fig. 6C). We did not observe significant differences among sperm populations (n=3, Mann-Whitney U-test, p>0.05).

As previous studies 38, 39 evidenced some proximity of telomeric chromosome extremities in spermatozoa and suggested that deregulation of chromatin architecture could result in increased intertelomeric distance, we also investigated this in chromosome 9 (Fig. 6D). We built home-made sub-telomeric probes and evaluated the distances between signals inside each nucleus, but again, no statistical differences were detected among the four sperm populations (n=3, Mann-Whitney U-test, p-values non-significant). Finally, we investigated the position of chromocenters among the populations (Fig. 6E) who appeared to be statistically similar (Table 5).

### CYTO-D treatment restores low-quality spermatozoa histone acetylation rate

**Figure 7:**
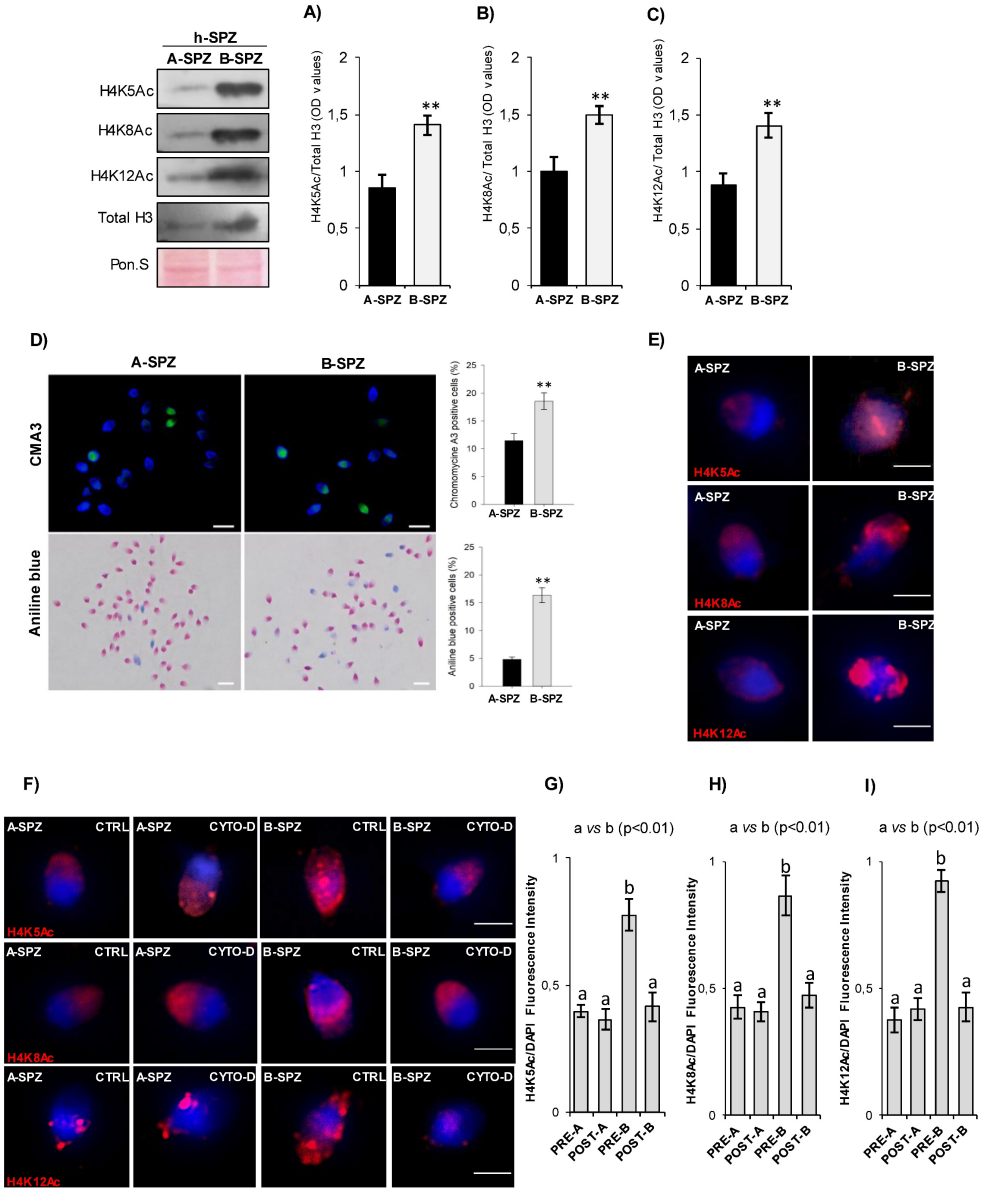
** F-actin depolymerization restores histone acetylation grade of low-quality spermatozoa.** (**A-C**) Western blot analysis of H4K5Ac (**A**), H4K8Ac (**B**) and H4K12Ac (**C**) in A- and B-SPZ fractions (n=5 different samples for each experimental group in triplicate). H4ac signals were quantified by densitometry analysis and normalized to total histone H3. Data were expressed in OD values and reported as mean ± SEM; **p < 0.01. (**D**) CMA3 and Aniline blue staining of A- and B-SPZ. Percentage values were reported as mean ± SEM; **p<0.01. Scale bar corresponds to 10 μm. (**E**) Immunofluorescence analysis of H4K5Ac (red), H4K8Ac (red) and H4K12Ac (red) in A- and B-SPZ fractions (n=6 samples for each experimental group). Nuclei were labeled with DAPI (blue); Scale bar correspond to 3 μm. (**F**) Immunofluorescence analysis of H4K5Ac (red), H4K8Ac (red) and H4K12Ac (red) in A- and B-SPZ fractions before (pre-A; pre-B) and following *in vitro* CYTO-D treatment (post-A; post-B) (n=6 samples for each experimental group); nuclei were labeled with DAPI (blue). Scale bar correspond to 3 μm. (**G-I**) Histogram showing the quantification of H4K5Ac (**G**), H4K8Ac (**H**) and H4K12Ac (**I**) signal intensity using ImageJ software. Values were expressed as mean ± SEM. Experimental groups with statistically significant differences (p<0.01) were indicated with different letters; the experimental groups without statistically significant differences were indicated with the same letter.

Since chromosome territory modulations were not responsible for the anomalous size of B-SPZ nuclei, as well as of nuclear resize occurred following CYTO-D treatment, we decided to investigate chromatin acetylation rate in sperm nuclei as histone acetylation is canonically associated with a more open and laxer chromatin structure. Firstly, we carried out a molecular and morphological characterization of total histone content and several histone H4 acetylation markers (H4K5Ac, H4K8Ac and H4K12Ac) between A- and B-SPZ by western blot analysis. As showed, an increased content of total histone H3, not dependent on protein amount variations, was observed in B-SPZ. Based on these data, western blot signals of histone H4 acetylation markers were normalized against total histone H3, in order to exclude any interference dependent on B-SPZ histone retention. Relatively to A-SPZ, and independently of histone retention, results showed a significant increase of all H4 acetylation markers in B-SPZ (p<0.01) (Fig. 7A-C), thus reinforcing our idea that spermatic chromatin acetylation rate could be the key factor involved in the anomalous size of B-SPZ nuclei. Chromomycine A3 (CMA3) and Aniline blue staining were carried out to assess protamine and histone content in A- and B-SPZ, respectively. In accordance with western blot data, a significant increase (p<0.01) in the percentage of positive cells, in each assay performed, was observed in B-SPZ (Fig. 7D), confirming histone retention in low-quality sperm population. Morphological characterization of histone H4 acetylations was performed by immunofluorescence analysis. A well-defined H4K5Ac and H4K8Ac localization was observed in the half anterior part of A-SPZ nuclei in comparison to a more intense and diffused signal occurred in B-SPZ (Fig. 7E). Interestingly, H4K12Ac signal was related to both anterior and posterior part of A-SPZ, in contrast to a diffused and disorganized distribution observed in B-SPZ (Fig. 7E), suggesting that an anomalous histone acetylation dynamic could be responsible of the increased nuclear size of B-SPZ.

Thus, with the aim to investigate if CYTO-D treatment preferentially induced B-SPZ nuclear resize acting on histone acetylation, we carried out immunofluorescence analysis of histone H4 acetylation markers on CYTO-D treated sperm cells (post-A; post-B) and on related control ones (pre-A; pre-B). Data confirmed that CYTO-D treatment did not affect immunolocalization of all acetylation markers in A-SPZ, whereas in B-SPZ (post-B) a clear physiological redistribution occurred (Fig. 7F). In addition, fluorescence intensity analysis confirmed H4K5Ac, H4K8Ac and H4K12Ac overexpression in B- when compared to A-SPZ, as previously observed, but more interestingly the intensity of signals of all acetylation markers in B-SPZ was restored to physiological A-SPZ values following CYTO-D treatment (Fig. 7G-I), suggesting that F-actin depolymerization induced nuclear resize of B-SPZ acting on histone acetylation rate and, in turn, on chromatin folding. Thus, the nuclear resize observed in post-B SPZ exclusively occurred following chromatin acetylation landscape dynamics dependent on nuclear F-actin modulation.

## Discussion

Epididymal transit consists of several well‑orchestrated and highly coordinated biological events which ensure the production of mature SPZ having egg-fertilization ability. This intricate epididymal network consists of maturational events acting on: i) cytoskeletal structure, ii) nuclear compaction and iii) RNA/protein payload, in order to provide SPZ every component needful for the fertilization process 40-42. In this scenario, acrosomal glycoprotein IZUMO1 represents a central actor in sperm-oocyte fusion, since necessary to the sperm-oocyte plasma membrane interaction; thus, male mice harboring IZUMO1 null mutation are completely infertile 10-11. In addition, testis-specific serine kinase TSSK6 regulates IZUMO1 phosphorylation and its acrosomal redistribution ensuring a sperm structural integrity correlated with the proper IZUMO1 localization useful for sperm‑egg fusion 12, 13.

Until today, the functional interplay among IZUMO1, TSSK6 kinase and actin dynamics have not yet been investigated in human SPZ and, even more interestingly, not linked to nuclear and chromatin sperm quality parameters. Based on these observations, in the current work, we carried out a comparative study of IZUMO1 in human high-quality spermatozoa (A-SPZ) and low-quality spermatozoa (B-SPZ), collected from normozoospermic volunteers, providing new findings regarding an intriguing IZUMO1 functional regulation exerted by nuclear F-actin. The molecular and morphological F-actin analysis carried out in A- and B-SPZ showed increased F-actin levels in B-SPZ, preferentially in sperm head, associated with a severe anomalous IZUMO1 delocalization in the posterior part of sperm head, relatively to sperm neck-midpiece section, strongly dependent on F-actin-IZUMO1 physical interaction. Considering that actin represents the most abundant cytoskeletal protein in the sperm head which actively regulates sperm morphology, the correct positioning of acrosome and its biological functions 1, 2, 8, our data led us to think at nuclear F-actin as a positive regulator of IZUMO1 positioning. With this in mind, we set an *in vitro* CYTO-D experiment on A- and B-SPZ, demonstrating that the induction of nuclear F-actin depolymerization in B-SPZ, effectively restored the physiological IZUMO1 acrosomal localization. Interestingly B-SPZ not only were characterized by the anomalous IZUMO1 neck-midpiece localization, but in addition showed a similar localization for the peanut lectin PNA antigen. Since PNA specifically stained acrosomal glycoproteins, we highlighted the idea that B-SPZ showed acrosome positioning anomalies dependent on an aberrant nuclear F-actin, as confirmed by CYTO-D treatment which restored the typical PNA anterior head localization. Furthermore, nuclear F-actin dynamics seem to drive sperm physiological functions, indeed the depolymerization of nuclear F-actin in B-SPZ favors a correct repositioning of the acrosome aimed at a successful AR onset, as demonstrated by CYTO-D treatment. Indeed, our data highlighted a new fascinating molecular mechanism, in which not only nuclear F-actin drives the correct pro-acrosomic vesicles movement in order to favor acrosome positioning and, in turn IZUMO1 localization, but also provides structurally conditions useful for AR onset.

In this scenario, TSSK6 analysis could not be excluded since its primary role in acrosomal IZUMO1 redistribution has been well reported 12, 13. Consistently with Li and coworkers 43, physiological human sperm TSSK6 localization was confined in a compartment posterior to the equatorial segment, whereas it was completely opposed in B-SPZ, thus appearing at acrosomal level. More interestingly, once again, CYTO-D treatment restored the normal TSSK6 pattern, further confirming the central role of F-actin in this intricate network. At this point to reinforce the idea that B-SPZ anomalies were dependent on F-actin dynamics linked to a spermatic immature state, we carried out a fine characterization of F-actin, IZUMO1 and TSSK6 in murine SPZ during their epididymal maturation, with the aim to correlate the immature *caput* SPZ murine phenotype with the human B-SPZ one. Accordingly, *caput* SPZ showed higher F-actin content in sperm head similar to B-SPZ, associated with acrosomal TSSK6 and diffuse IZUMO1 localizations. More interestingly, during epididymal transit the depolymerization of F-actin related to sperm head affected actin-IZUMO1 physical interaction to favor TSSK6 posterior translocation and, in turn, the prominent acrosomal IZUMO1 localization dependent on a massive phosphorylation rate acquisition. All together these findings are consistent with the recent literature which have reported: i) IZUMO1 phosphorylation increase and redistribution during epididymal sperm maturation of rat SPZ, ii) posterior TSSK6 localization in both *cauda* murine SPZ and human ejaculated SPZ 12, 13, 41.

In the current work we suggest that nuclear F-actin and its polymerization dynamics are very important in the regulation of TSSK6-IZUMO1 pathway. Accordingly, depolymerization of nuclear F-actin in B-SPZ, induced by CYTO-D treatment, perfectly matched with murine epididymal transit data, since the loss of F-actin-IZUMO1 interaction specifically enhanced IZUMO1 phosphorylation, reinforcing, and thus confirming, that low-quality B-SPZ reflected the immaturity grade of murine *caput* SPZ.

Considering that nuclear actin compartmentalization regulates chromosome movement and positioning as well as chromatin remodeling, we asked if aberrant nuclear F-actin related to low-quality sperm population could affect chromosomal and chromatin parameters, additionally to the effects described on IZUMO1 localization 15, 17-19. To answer this question, we analyzed CYTO-D responsiveness of sperm nuclear parameters starting from a “macroscopic nuclear study”, up to zooming towards a “microscopic analysis” focused on chromatin folding. Surprisingly, not only B-SPZ showed abnormal nuclear size parameters, but nuclear F-actin depolymerization was able to restore nuclear size, in term of area and perimeter, towards physiological values. Based on these enthusiastic results, we focused our attention on a possible chromosomal territory movement responsible of nuclear remodeling and correlated with nuclear F-actin dynamics. Interestingly, chromosomal territories as well as inter-telomeric distances were not affected in B-SPZ, excluding that F-actin dynamics regulated sperm nuclear size *via* the modulation of chromosome positioning. Overall, sperm nuclear architecture of sperm populations here analyzed did not appear significantly different, even if it is possible that modifications went unnoticed because of the limited bidimensional character of our experiments.

Since the production of functional spermatozoa is highly dependent on regulated gene expression pattern, which in turn is tightly linked to epigenetic landscape, putative histone modifications-driven chromatin folding changes in B-SPZ were not excluded 5, 44. Effectively, B-SPZ showed a high histone retention, but more interestingly an increased chromatin acetylation grade, not dependent on histone retention, was completely reverted following F-actin depolymerization induction. Our data are consistent with recent findings highlighting the role of nuclear actin in chromatin remodeling complexes activity, able to modulate histone acetyltransferase activity *in vitro* and *in vivo* 45. In addition, nuclear actin polymerization dynamics actively modulate histone acetylation *via* interaction with histone de-acetyltransferases (HDACs) that in turn allow chromatin remodelers and histone acetyltransferases (HATs) in order to relax chromatin topology into a transcription-ready state 46. It cannot be excluded that a similar molecular regulation occurs in sperm cells and that the depolymerization of nuclear F-actin, induced by CYTO-D treatment, activates specific chromatin remodeler complexes driving histone de-acetylation and, therefore, chromatin compaction responsible for B-SPZ nuclear reduction. A similar behavior has been demonstrated in daughter cells after cell division where the removal of actin polymerization within the nucleus results in increased chromatin compaction in daughter nuclei 47.

Overall, the results obtained demonstrate that nuclear F-actin affected IZUMO1 localization and spermatic nuclear size *via* histone acetylation regulation. In addition, we have identified, for the first time, F-actin-IZUMO1 physical interaction highlighting: i) F-actin polymerization dynamic role on TSSK6 activity and, in turn, on IZUMO phosphorylation, occurring along the epididymal transit, ii) the identification of IZUMO1 as a potential morphological marker useful to select sperm cells characterized by morphological as well as molecular and chromatin good parameters and iii) nuclear F-actin role in the regulation of acrosome positioning and AR onset.

## Figures and Tables

**Table 1:** Sperm motility parameters in human high- and low-quality spermatozoa.

	A-SPZ	B-SPZ
Progressive (%)	85.16±0.02	9.09± 0.07**
VCL (µm/s)	52.34±0.05	26.01±0.14**
VSL (µm/s)	33.53±0.09	14.13±0.10**
VAP (µm/s)	38.11±0.09	17.17±0.06**
LIN (%)	66.39±0.07	54.32±0.11*
STRA (%)	87.97±0.06	79.01±0.08*

**: p<0.01; *: p<0.05

**Table 2:** Sperm motility parameters in human high- and low-quality spermatozoa following CYTO-D treatment.

	Pre-A	Post-A	Pre-B	Post-B
Progressive (%)	82.67±0.12	11.46±0.11**	8.69± 0.08	5.21± 0.08*
VCL (µm/s)	50.14±0.04	36.04±0.09**	23.01±0.11	15.18±0.11**
VSL (µm/s)	33.87±0.10	23.63±0.11**	15.23±0.12	10.83±0.12**
VAP (µm/s)	39.01±0.13	27.02±0.08**	16.56±0.09	9.82±0.09**
LIN (%)	69.21±0.08	57.45±0.09*	51.72±0.10	44.29±0.10*
STRA (%)	89.43±0.08	78.33±0.10*	78.56±0.07	68.36±0.07*

**: p<0.01; *: p<0.05

**Table 3:** Pre-B spermatozoa display a statistically higher area than other sperm populations.

	Pre-A	Pre-B	Post-A	Post-B
Area median ± SEM	76.94 ± 0.53	81.64 ± 0.57	78.36 ± 0.41	77.92 ± 0.50
Area mean 95% CI	76.54 ± 1.05	81.38 ± 1.13	77.70 ± 0.80	77.91 ± 0.98
Area coefficient of variation	0.15	0.13	0.13	0.14
Perimeter median	34.14 ± 0.13	35.42 ± 0.17	34.34 ± 0.11	34.39 ± 0.13
Perimeter mean 95% CI^	34.13 ± 0.26	35.50 ± 0.33	34.42 ± 0.21	34.58 ± 0.26
Perimeter coefficient of variation	0.08	0.09	0.08	0.08

^CI= Confiance Interval

**Table 4:** Statistical data associated to Figure 4A.

	Area		Perimeter	
	Mann-Whitney U statistics	p-values	Mann-Whitney U statistics	p-values
Pre-A *vs* Pre-B	93027	<0.0001	94258	<0.0001
Pre-A *vs* Post-A	145181	NS	143771	NS
Pre-A *vs* Post-B	110368	NS	113450	NS
Pre-B *vs* Post-A	128506.5	<0.0001	130549.5	<0.0001
Pre-B *vs* Post-B	96599	0.0001	95044	0.0008
Post-A *vs* Post-B	150553	NS	156288.5	NS

NS= Non-Significant

**Table 5:** Statistical data associated to Figure 4E.

Groups	Luminance	Contrast	Structure	MS-SSIM^
Pre-A *vs* Pre-B	0.999	0.929	0.834	0.774
Pre-A *vs* Post-A	0.998	0.939	0.846	0.793
Pre-A *vs* Post-B	0.999	0.918	0.862	0.791
Pre-B *vs* Post-A	0.998	0.930	0.845	0.783
Pre-B *vs* Post-B	0.99	0.937	0.847	0.793
Post-A *vs* Post-B	0.996	0.902	0.840	0.754

^MS-SSIM= multi-scale structural similarity index
